# Prostate MRI: Is Endorectal Coil Necessary?—A Review

**DOI:** 10.3390/life12040569

**Published:** 2022-04-11

**Authors:** Grace Lee, Aytekin Oto, Mihai Giurcanu

**Affiliations:** 1 Department of Radiology, University of Chicago, 5841 South Maryland Avenue, Chicago, IL 60637, USA; aoto@radiology.bsd.uchicago.edu; 2 Department of Public Health Sciences, University of Chicago, 5841 South Maryland Avenue, Chicago, IL 60637, USA; giurcanu@uchicago.edu

**Keywords:** endorectal, staging, detection, signal to noise ratio, wearable perineal coil

## Abstract

To assess the necessity of endorectal coil use in 3 Tesla (T) prostate magnetic resonance imaging (MRI), a literature review comparing the image quality and diagnostic performance with an endorectal coil (ERC) and a without endorectal coil (NERC), with a phased array coil or a wearable perineal coil (WPC), was performed. A PubMed search of 3T prostate MRI using an endorectal coil for studies published until 31 July 2021 was performed. A total of 14 studies comparing 3T prostate MRI with and without endorectal coil use were identified. The quality scores and diagnostic performances were recorded for each study. In total, five studies compared image quality; five studies compared quality and performance; and four studies compared performance of detection, size of detected lesions, accuracy of cancer localization, and aggressiveness/staging. The use of an endorectal coil improved image quality with a higher overall signal to noise ratio, posterior and peripheral zone signal to noise ratio, high b-value attenuation diffusion coefficient (ADC) signal to noise ratio, and contrast to noise ratio. Endorectal coil use improved subjective image quality for anatomic detail on T2 weighted images (T2WI) and diffusion weighted images (DWI). Endorectal coil use had less motion artifact on DWI than non-endorectal coil use, but produced a higher occurrence of other artifacts on DWI. Endorectal coils had higher sensitivity, specificity, and positive predictive value (PPV) in the detection of overall and index lesions, as well as smaller and less aggressive lesions, missing fewer and smaller lesions than non-endorectal coils. Endorectal coils had higher sensitivity than non-endorectal coils in localizing and staging lesions. Endorectal coils improved quantitative and qualitative image quality and diagnostic performance in the detection of smaller and less aggressive cancers in 3T prostate MRI.

## 1. Introduction

Prostate MRI has become instrumental in diagnosing prostate cancer, guiding biopsy in patients with elevated prostate specific antigen (PSA), and in local staging of prostate cancer. Advances in technology have yielded high sensitivity and negative predictive values of up to 96% sensitivity [1] and negative predictive value (NPV) of 68–100% [2,3] for the diagnosis of prostate cancer. Moreover, multiparametric MRI (mpMRI) contributes to risk stratification in distinguishing Gleason 3 + 3 from Gleason scores greater than 6.

**Figure 1:**
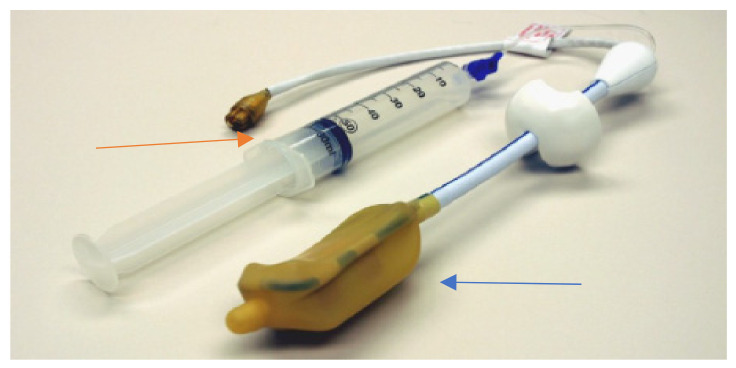
Medrad endorectal coil, courtesy of University of Chicago. Blue arrow: inflatable, disposable endorectal balloon. Orange arrow: 60 cc air-filled syringe.

With the widespread use of 3T mpMRI in the community and in academia, reproducible diagnostic performance becomes paramount. One conspicuous variable among exams is the use of Endorectal coils (ERC). ERC may be mandatory for 1.5T imaging, but there are no clear recommendations at 3T [4,5]. ERC is infrequently utilized in community radiology as well as in academic radiology, with only 30% of academic radiology centers instituting Endorectal coils for 3T exams [4]. Guidelines for quality imaging and standardized interpretation with Prostate Imaging Reporting Archiving Data Systems (PIRADS) version 2.1 have been implemented to reduce inconsistency in the exams. The disadvantages of ERC use are patient discomfort (Figure 1), technologists’ time, and coil cost. ERC unquestionably increases the signal to noise ratio and improves spatial resolution, but its essential contribution to quality and diagnostic performance is challenged. MR H Spectroscopy, originally included in PIRADS version 1, has been omitted from PIRADS version 2, despite its good positive predictive value and high negative predictive value in distinguishing prostate cancer from prostatitis, and is limited by the need to monitor the quality of automated post-processing and documentation of steps taken. Dynamic contrast enhancement (DCE) provides the added signal via contrast, but as the trend towards biparametric imaging gains momentum, the role of diffusion weighted imaging (DWI) becomes more crucial. Additionally, quality monitoring becomes more important to these sequences which are most susceptible to artifacts. Future efforts to improve detection and accurate staging of prostate cancer with high channel coils to enhance signal and resolution, as well as emerging methods such as deep learning, may obviate the need for the endorectal coil.

## 2. Materials and Methods

Literature search: A PubMed electronic search for studies on endorectal prostate MRI at 3T published until 31 July 2021 was utilized.

Eligibility Criteria: The studies that were selected compared prostate MRI on 3T for prostate cancer without prior treatment, utilized an endorectal coil, compared quality and/or diagnostic performance, had pathologic correlation by MRI fused transrectal ultrasound guided biopsy or prostatectomy, and employed T2 weighted and DWI sequences. Reviews and editorials were excluded.

Information Sources: PubMed.

Search Strategy: An electronic search of PubMed for MRI Prostate, 3T, comparison, and endorectal coil was performed. The search was limited to human patients or phantoms, and published in English.

Inclusion Criteria: The studies were chosen if they met the following criteria: treatment naïve, clinically suspected or biopsy-proven patients undergoing 3T MRI; compared prostate MRI with and without an endorectal coil; used at least T2 weighted imaging.

Exclusion Criteria: The studies were excluded if they met the following criteria: compared with 1.5T; were review articles, guidelines, or editorials; did not use endorectal coil, did not concern prostate cancer.

Data Extraction and Quality Assessment: The following characteristics were assessed: study characteristics, design, single or multicenter study, prospective versus retrospective, pathologic reference standard, patient characteristics of number of patients, age, interval between prostate biopsy and MRI and MRI and surgery, PSA level, Gleason scores were recorded; imaging traits of magnet strength and coil type, sequences, image plane, matrix, slice thickness, the number of excitations were recorded; reader characteristics of number of readers, experience of readers, type of read as independent or consensus, whether readers were blinded to the histopathology were recorded.

Selection Process: One reviewer screened the studies without the use of automation tools.

Data Collection Process: One reviewer screened the studies without the use of automation tools.

Outcome Assessment: The outcomes assessed were: the objective image qualities of signal to noise ratio, contrast to noise ratio, and integral uniformity; subjective image qualities of anatomic detail for localization and tissue distinction, motion, and other artifacts; and the diagnostic performance of prostate MRI using an endorectal coil (ERC) and prostate MRI using a non-endorectal coil (NERC) with either phased array or a wearable pelvic coil in the detection, localization, and staging of prostate cancer.

## 3. Results

### 3.1. Literature Search

Endorectal coil MRI prostate = 495

3T = 126

Comparison = 31

Excluded: Segmentation = 5

Spectroscopy = 5

T2 maps = 1

Post prostatectomy = 1

Radiation therapy = 3

Positron Emission Tomography (PET) = 1

Prostate with benign prostatic hyperplasia = 1

Remaining = 14

### 3.2. Study Characteristics

There were 14 studies [6,7,8,9,10,11,12,13,14,15,16,17,18,19] on 3T that compared ERC to NERC, including three studies that compared ERC to the wearable perineal coil (Ha et al., O’Donohue et al., Ullrich et al.). A total of 10 studies were conducted with ERC and NERC in the same sitting, three were not conducted in this way (Ullrich et al., Mirak et al., Kim et al.), and one was with or without the same sitting (Gawlitza et al.).

Of the 14 studies, seven were prospective [6,7,11,12,13,17,18]. A total of four studies were retrospective [9,16,18,19] (Mirak et al. was prospective, then retrospective). Overall, four studies did not specify whether they were prospective or retrospective. In total, five studies [7,8,9,10,13] evaluated image quality items of signal to noise ratio, contrast to noise ratio, integral uniformity, T2WI quality contributing to anatomic detail and zone distinction, DWI quality to visualize anatomy, motion on T2WI and DWI, and other artifacts of geometric gland distortion, blurring, ghosting, flare signal, wrapping, and susceptibility.

A total of four studies evaluated diagnostic performance [6,15,16,18] of sensitivity, specificity, and PPV of detection of overall cancers; sensitivity, specificity, and PPV of detection of index cancers; and accuracy of the localization of cancer, and accuracy of staging of cancer by extra-prostatic extension or seminal vesicle involvement.

Overall, five studies evaluated image quality and diagnostic performance [11,12,14,17,19].

A total of two studies utilized DCE [16,18].

For more information, see Table 1: Study Characteristic and Table 2: MRI Parameters.

### 3.3. Patient Characteristics

The range of number of patients within each study was from 20–429. The range of mean or median age was from 60–66 years old, and if the median was not given, the range was 49 to 79 years old. The range in PSA median was 6.3–14.1 ng/mL. If a median was not given, the range was 2.5–48.3 ng/mL. A total of seven studies submitted a Gleason score mean or range of 1–9. Overall, four studies listed the range of time of exam to surgery between 2.2 days to 133 days.

For more information, see Table 3: Patient Characteristics and Table 4: Gleason Score, Location, and Presence/Absence of Extra-prostatic Extension.

### 3.4. Index Test Characteristics

A total of nine studies compared ERC with NERC in the same sitting [6,7,10,11,12,13,15,16,17], and seven studies were conducted with NERC followed by ERC. The two Barth et al. studies were conducted with ERC followed by NERC [13,16]; four studies were not conducted in the same sitting [9,14,18,19]. Overall, eight studies utilized pathologic confirmation with radical prostatectomy [10,11,13,14,15,16,18,19] as the standard of reference. A total of two studies utilized transrectal ultrasound-guided biopsy as the standard of reference [9,12]. One study used both [17], and one study had a mixed cohort of radical prostatectomy and transrectal ultrasound-guided biopsy as the standard of reference [6].

### 3.5. Reader Characteristics

The range of number of readers was 1–6, and the range in experience of readers was several months to 18 years. Overall, four studies blinded the readers [14,15,16,18]. For more information, see Table 5: Index Test Characteristics.

### 3.6. Quality Characteristics

The studies evaluated objective qualities of signal to noise ratio (SNR), contrast to noise ratio (CNR) between the prostate gland versus the biopsy-proven prostate cancer, CNR between the transition zone versus peripheral zone, and integral uniformity. Subjective qualities of zonal anatomy distinction, T2WI and DWI anatomic conspicuity, motion, geometric distortion, and other artifacts of susceptibility, blurring, ghosting, flare signal, and wrapping were rated on a Likert scale of 1 to 5. For more information, see Table 6: Outcomes Measured.

### 3.7. Diagnostic Performance Characteristics

The diagnostic performance items of sensitivity, specificity, PPV, the area under the receiver operator characteristics curve (AUC) for the detection of the overall and index cancer, the maximum diameter of the lesion detected, the accuracy of identification of the location of cancer, the accuracy of detection of a low-grade cancer, the accuracy of extra-prostatic extension of cancer by detection of narrowed rectoprostatic angle, capsule penetration, and seminal vesicle invasion were evaluated.

## 4. Discussion

Quality was evaluated objectively, with quantifiable parameters of overall signal to noise ratio, regional SNR, and high b-value ADC SNR. Integral uniformity was also measured. Other parameters measured were contrast to noise ratio between the whole prostate and the cancer, and CNR between the transition zone and peripheral zone. ERC provided higher SNR and CNR, but lower integral uniformity. Subjective image quality was scored by readers on a Likert scale of 1 to 5, rating items on distinction of zonal anatomy, motion, and other artifacts.

**Figure 2:**
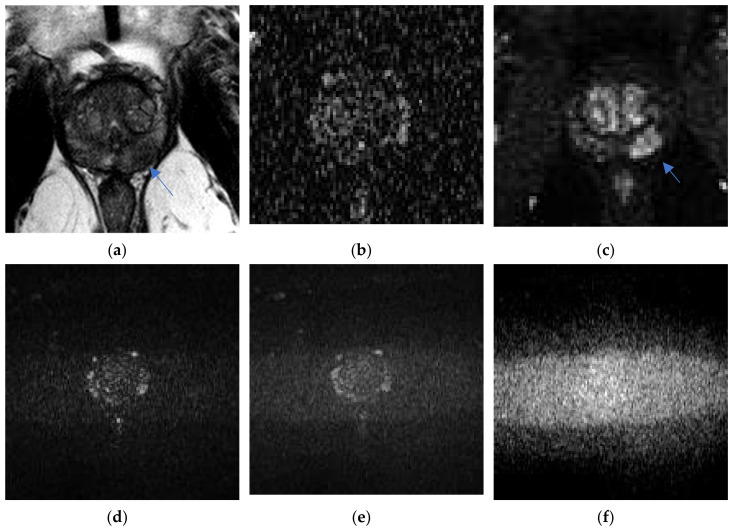
Poor signal to noise with non-endorectal coil (NERC) on high b-value DWI: a 61-year-old man, PSA = 7.8 ng/mL, Gleason = 4 + 3 left mid-gland peripheral zone, cribriform pattern with extra-prostatic extension, only detected (arrow) as T2 hypo-intensity on Philips Achieva 3T NERC T2WI (**a**) and early enhancement on NERC DCE (**c**), undetected on NERC ADC (**b**) and NERC b = 50 s/mm^2^ (**d**), b = 150 s/mm^2^ (**e**), and high b-value = 990 s/mm^2^ DWI (**f**). Images courtesy of University of Chicago.

The ERC improved quality by providing enhanced signal to the most crucial, but most signal-deprived sequence, DWI. Studies comparing the ERC to NERC, as well as the two studies by O’Donohue et al. and Ullrich et al. comparing ERC to WPC, demonstrated higher SNR with the ERC, which was most beneficial to DWI. ERC also increased the SNR for T2 weighted images (T2WI), see Figure 2.

All studies comparing signal to noise ratios demonstrated that ERC provided a higher overall SNR compared to phased array non-endorectal coils (NERC) at 3T. ERC supplied an SNR which was two times greater to T2WI, or 14.75+/−3.92 versus 11.53+/−3.44 [6,19], and an SNR which was 1.7 times greater to DWI [6]. Barth et al. [13] found higher ERC SNR more specifically on DWI: ERC DWI SNR = 26.9 versus NERC DWI SNR = 19.2 in the whole prostate; and in the peripheral zone, ERC DWI SNR = 28.1 versus NERC DWI SNR = 20.5, although Barth et al. (2016) concluded that NERC provided equivalent image quality to ERC. Kim et al. [19] found higher ERC = T2WI SNR 14.75+/−3.92 versus NERC T2WI SNR = 11.53+/−3.44 (*p* = 0.081), but concluded it was not statistically significant. In comparison to the more recently developed wearable perineal coils (WPC 8 or 16 channel), ERC maintained a superior SNR for T2WI with ERC T2WI SNR = 38.3 versus WPC T2WI SNR = 16.6; and a greater ERC DWI(b-1400 s/mm^2^) SNR = 81.5 versus WPC DWI SNR = 19.8 versus NERC DWI SNR = 22.8 [7] (*p* < 0.001), confirming the results of an earlier study of a five channel WPC, finding that the ERC SNR heterogeneous value = 291–684 was 8% higher than the WPC SNR (=459–470), compared to NERC (=146–159) [8].

Regionally, ERC provided a higher DWI SNR in various anatomic locations, such as the peripheral zone, a location frequently containing prostate cancers, as concluded by Barth et al. [13]. Comparing ERC to WPC, ERC provided a higher SNR than WPC in the anterior prostate peripheral zone, with ERC T2WI SNR = 6.2 versus WPC T2WI SNR = 4.4. In the anterior transition zone, ERC SNR T2WI = 8.3 versus WPC SNR T2WI = 5.1. ERC provided a higher SNR compared to the WPC in the whole prostate: ERC SNR = 59.3 versus WPC SNR = 33.9. ERC provided a higher SNR in the peripheral zone (PZ), ERC SNR = 76.7 versus WPC SNR = 33.9. ERC had a higher SNR in the transition zone (TZ), ERC SNR = 52.5 versus WPC SNR = 34.9. ERC had a higher SNR in the prostate cancer lesion, ERC SNR = 83.2 versus WPC SNR = 44.8 [9].

The high b-value ADC SNR also was higher with ERC. The high b-value ADC ERC SNR was also higher in the peripheral zone, a crucial sequence and location, increasing the detection of cancer. The ADC is underestimated at a lower SNR. The ADC SNR decreases at higher b-values. The underestimated ADC also occurs at higher true ADC values. The SNR, and consequently the estimated ADC, was less degraded in the PZ. The ERC high b-value ADC SNR in the PZ was 9.27 times higher than the NERC high b-value ADC SNR; the ERC high b-value ADC SNR in the TZ was 5.5 times higher than the NERC high b-value ADC SNR in the TZ, with the difference progressively greater on the high b-value sequences [10].

Although the SNR of the ERC of 150–710 was 8% higher than the SNR WPC, the higher ERC integral uniformity (IU) parameter for T2WI and DWI caused lower clarity and more artifacts, outlined in an earlier study [8] that compared the WPC IU = 1.2% to the NERC IU = 7.8% and the ERC IU = 40.4%. Higher values indicated greater heterogeneity, but the study did not compare the diagnostic performance.

Ullrich et al. [9] demonstrated a higher ERC contrast to noise ratio than the WPC. ERC provided an increased contrast to noise ratio for T2WI and DWI due to the closer proximity of the ERC coil to the prostate. ERC had a higher contrast to noise ratio (CNR) compared to the WPC, between the lesion and the prostate with ERC CNR = 18.82 versus WPC CNR = 8.85. ERC also had a higher CNR between the peripheral zone and transition zone with ERC CNR = 24.25 versus WPC CNR = −0.94 [9].

Most studies provided variable results in subjective quality comparisons between the ERC and NERC. The subjective quality ratings were either equivalent between ERC and NERC for the T2WI, and demonstrated worse performances in other artifacts, such as distortion with the ERC. The WPC quality ratings were higher for T2 weighted images.

**Figure 3:**
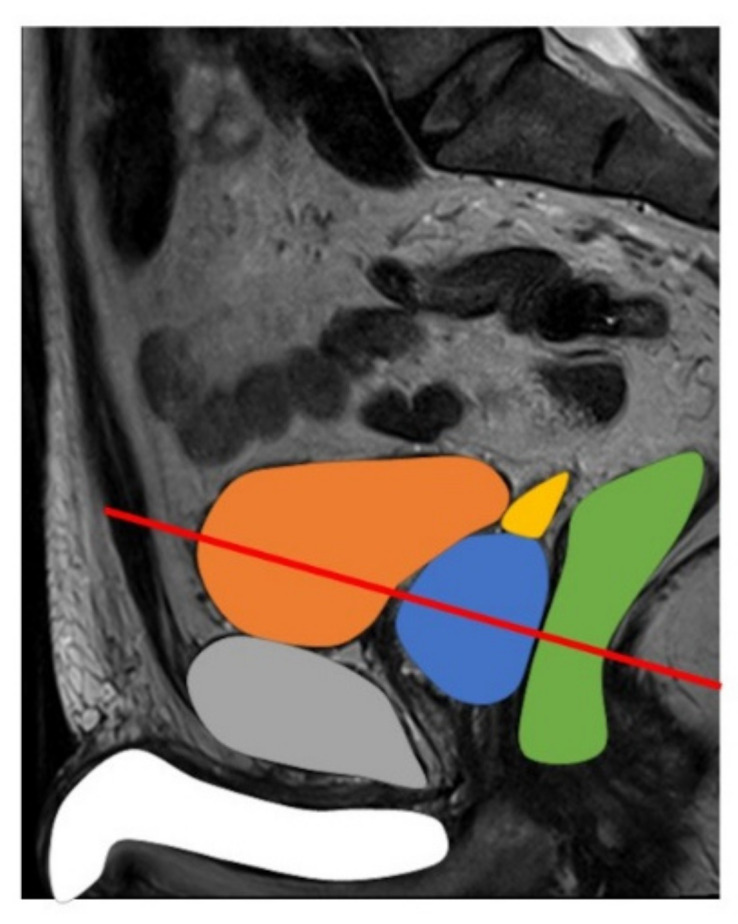
Sagittal view of male pelvis on MRI: bladder–orange, prostate–blue, seminal vesicles–yellow, rectum–green, pubic bone–grey, penis–white. Red line indicates plane of image with the endorectal coil located in the rectum.

Studies comparing image quality of ERC versus NERC rated zonal anatomy to be better visualized with ERC. Clear visualization of anatomy may be degraded by the close proximity of the prostate to the rectum or bladder, see Figure 3. ERC usually had an equivalent or higher subjective quality score on a scale of 1–5, when rated by multiple readers and studies, in evaluating distinct zonal anatomy, implying better subjective quality ratings for T2WI. ERC DWI consistently produced higher anatomic quality scores. Image quality scores on a scale from 1 to 5 for anatomic distinction (5 rated for the best) rated higher scores for ERC: ERC = 4.1 versus NERC = 3.1, as well as overall image quality scores for ERC = 4.03 versus overall image quality scores for NERC = 3.18 [11].

ERC T2WI (45 exams) versus NERC T2WI (23 exams) had a greater number of excellent overall excellent quality exams for anatomy; ERC DWI (19 exams) had a greater number of excellent quality exams for anatomy versus NERC DWI (16 exams) [12].

One study had equivalent two-reader anatomic quality scores for ERC T2WI = 3.07, 3.77 compared to NERC T2WI = 3.27, 3.70, but used higher NEX NERC T2WI; however, there were higher anatomic quality scores for ERC DWI = 3.08, 3.66 versus NERC DWI = 3.03, 3.27 [13].

ERC anatomy quality scores were variable in comparison to WPC on T2WI, but consistently superior for ERC DWI. The ERC T2WI quality score was 3.94 versus WPC T2WI quality score = 3.83 [7], but only 17.8% of ERC T2WI were preferred, as compared to 38.7% WPC T2WI in another study [9]. For DWI, ERC DWI quality score = 4.28 was higher than WPC DWI quality score = 3.72 [7]; in another study,50.9% of ERC DWI exams were preferred to 19.6% WPC DWI exams [9].

Image quality studies also rated zone and lesion localization. ERC T2WI provided higher lesion localization scores compared with NERC T2WI. The ERC T2WI average score was 4.25 versus NERC T2WI average score = 3.4 for two readers [14].

Staging scores were higher with ERC versus NERC comparison studies, with the ERC quality staging average score = 4.4 versus NERC quality staging average score = 4.2 for 6/24 channel system [14]; and ERC score = 4.15 versus NERC score = 3.3 for an eight-channel system [11].

The qualitative evaluation of motion and other artifacts was variable. Most studies concluded that ERC displayed less motion, but more other artifacts: earlier studies showed that ERC had greater motion artifact with an ERC motion score = 2.76 versus NERC motion score = 1.51 (a lower score is better [11]). Recent studies concluded ERC had less motion due to the stabilizing presence of the coil on T2WI [12] and DWI [9]. Recent studies showed that a higher number of ERC T2WI exams had no motion = 46 exams versus NERC T2WI no motion = 31 exams [12]. For DWI motion, ERC had less motion compared to WPC: ERC DWI motion artifact score = 1.16 versus WPC DWI motion artifact score = 1.58 (lower motion score is better [9]). Most studies concluded there were more other artifacts, such as aliasing, ghosting, or blurring, on ERC T2WI compared with NERC T2WI or WPC T2WI, and on ERC DWI compared to NERC DWI. The higher frequency of other artifacts on ERC T2WI = 72 exams versus NERC T2WI = 49 exams were susceptibility, and flare at the coil interface on ERC DWI = 71 exams versus NERC DWI = 76 exams [12]. Another study found that ERC had a higher number of exams with artifact ERC = 109 versus NERC number of exams with artifact = 75 [13]; ERC had more additional artifacts for one reader. A second reader found that ERC had less susceptibility, ghosting, wrapping, and blurring [13]. Another study of two readers found ERC versus NERC artifacts of motion and others (susceptibility/aliasing) were equivalent [14].

When comparing T2WI artifacts on ERC versus WPC, the ERC T2WI artifact score = 2.01 versus WPC T2WI artifact score = 1.39 (worse) [7].

Most studies evaluated the two methods by comparing not only image quality, but also diagnostic performance. Diagnostic performance was analyzed by sensitivity, specificity, and positive predictive value of overall and index lesion detection, size of detected lesions, accurate localization of lesions, accurate grading of lesions, sensitivity, specificity, and accuracy of staging. Earlier studies demonstrated that ERC provided better diagnostic performance in the detection and staging. Heijmink et al., in 2007, studied 46 men at 3T with a standard of reference of whole mount pathology [11]. More recently, Turkbey et al. evaluated 20 patients at 3T with a standard of reference of whole mount pathology in 2014, and concluded that ERC detected smaller size cancer lesions [15]. ERC was more accurate in detecting the grade of lesion [6]; Costa et al. evaluated 49 patients at 3T ERC versus NERC versus DCE in 2016, and concluded ERC had a higher detection rate than DCE, which had a higher detection rate than NERC for significant cancers, with fewer false negative misses of high-grade lesions.

Most studies identified a higher sensitivity of detecting lesions with ERC: ERC sensitivity = 73–80% versus NERC sensitivity = 7–73%; however, the specificity of ERC = 94–97% versus NERC = 91–100% remained equivalent [11].

Overall lesion detection, index lesion detection, and PPV were higher with ER: overall lesion ERC sensitivity = 76% versus overall lesion sensitivity NERC = 45%; ERC index lesion sensitivity = 85% versus NERC index lesion sensitivity = 75%. ERC PPV = 0.80 versus NERC PPV = 0.64 [15].

Overall lesion detection and index lesion detection sensitivity were higher with ERC: ERC detection sensitivity = 78% versus NERC overall detection sensitivity = 43% [6], and ERC index lesion sensitivity = 92% versus NERC index lesion sensitivity = 47%, *p* < 0.01 [6]. The cancer miss rate was lower with ERC: ERC miss rate = 4% versus NERC miss rate = 42% *p* = 0.02 [6].

The detection of number of lesions was higher for ERC: ERC = 80% versus NERC = 73.5% [14].

ERC had a higher sensitivity and PPV for detecting intermediate and high-grade lesions (equal or greater than Gleason 3 + 4) in 33 patients between 2014–2015, with the standard of reference of whole mount pathology specimens: ERC sensitivity average = 0.82 versus NERC sensitivity average = 0.64; the ERC PPV = 0.89–0.91 versus NERC PPV = 0.80–0.81. On a per patient and per side basis, ERC sensitivity was higher: the total ERC sensitivity for lesion detection = 0.85–0.97 versus NERC sensitivity for lesion detection = 0.76–0.82. On a per side basis, ERC PPV = 0.94 versus NERC PPV = 0.87 [15].

Although Barth et al. (2019) [16] concluded equivalent detection of prostate cancer when comparing ERC with NERC at 3T, there were five and three false negatives by Reader 1 and 2, respectively, on NERC compared to ERC, which was attributed to lower SNR on NERC, despite the study’s application of increased NEX on NERC: NERC T2 NEX = 4 versus ERC T2 NEX = 3, NERC DWI NEX = 2,6,12 versus ERC DWI NEX = 2,4,8 in addition to increased signal provided with a lower NERC T2 matrix = 256 × 256 versus ERC T2 matrix = 320 × 320 and lower NERC DWI matrix = 100 × 52 versus ERC DWI matrix = 112 × 58.

Although Baur et al. [12] demonstrated no significant difference in sensitivity, AUC, PPV, and NPV between NERC and ERC, the cutoff score of Gleason score = 7 was the threshold for positive lesions, and the performance for detection of less aggressive lesions was not evaluated. In addition, the standard of reference was transrectal ultrasound-guided core biopsy, which would exclude the detection of false negative lesions. Despite the conclusion that there was no significant difference in the diagnostic performance of NERC versus ERC, the study did yield a higher ERC specificity = 0.74, 0.67 versus NERC specificity = 0.61, 0.54 for two readers.

**Figure 4:**
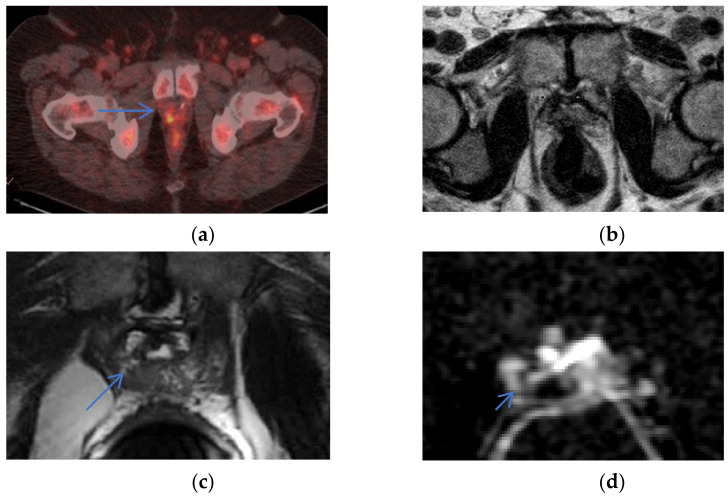
Recurrence not detected on NERC: a 71-year-old man with rising PSA after prostatectomy. Recurrence detected in the right pelvis (arrow) as hypermetabolic activity on Axumin/PET (**a**), undetected on NERC T2WI (**b**), and detected as T2 hypo-intensity in the right prostate bed on Philips Achieva 3 T ERC T2WI (**c**) and ERC ADC (**d**). Images courtesy of University of Chicago.

ERC can provide higher sensitivity to detect recurrence, see Figure 4.

Overall, three studies, Heijmink et al., Costa et al., and Baur et al., provided complete data to calculate a pooled sensitivity and specificity, indicating higher ERC performance than NERC. The pool is heterogeneous: Heijmink et al., had the largest sample, while Costa et al. and Baur et al. had smaller samples. Moreover, the sensitivity of Heijmink’s et al. data was low due to the use of T2 weighted images only, compared to the use of T2 weighted images and DWI for Costa et al. and Baur et al. The pooled ERC sensitivity = 0.754 (SE = 0.17); 95% CI: 0.326–0.946; NERC sensitivity = 0.645 (SE = 0.21); 95% CI: 0.231–0.916. The pooled ERC specificity = 0.77 (SE = 0.452); 95% CI: 0.0.675–0.85; NERC specificity = 0.742 (SE = 0.50), 95% CI: 0.633–0.828. The random effects of the odds ratio are: for diagnostic sensitivity = 2.36 variance; the diagnostic specificity = 0.176, and the correlation between the sensitivity and specificity = −0.9. The variance was higher for sensitivity than specificity. After removing the variation between the studies, the ERC/NERC sensitivity diagnostic odds ratio = 1.61 (SE = 0.182), *p* = 0.001. The ERC to NERC specificity diagnostic odds ratio = 1.20 (SE = 0.101) *p* = 0.341, which was less than 0.05. We fitted a mixed effects binomial regression for performance measures, with fixed effects for diagnostic type (positive/negative), type of MRI (ERC/NERC), and their interaction, with the study using random effects grouped by diagnostic type. This mixed effects binomial model specification allowed us to assess the sensitivity and specificity by MRI type, as well as to assess variability in sensitivity and specificity and their correlation between studies. Data analysis was conducted using the statistical software R (version 4.1.2) and R Studio, and the R package lme4 was used for the mixed effects binomial model fit. Forest plots and performance measures were calculated using the R package mada. An R markdown file with fully reproducible data analysis is available from the authors upon request.

**Figure 5:**
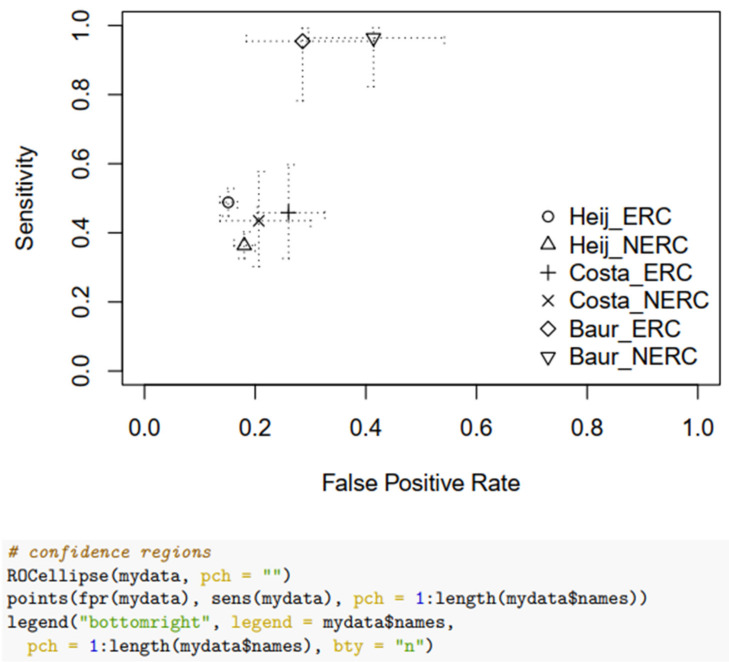
Forest Plot for Sensitivity.

**Figure 6:**
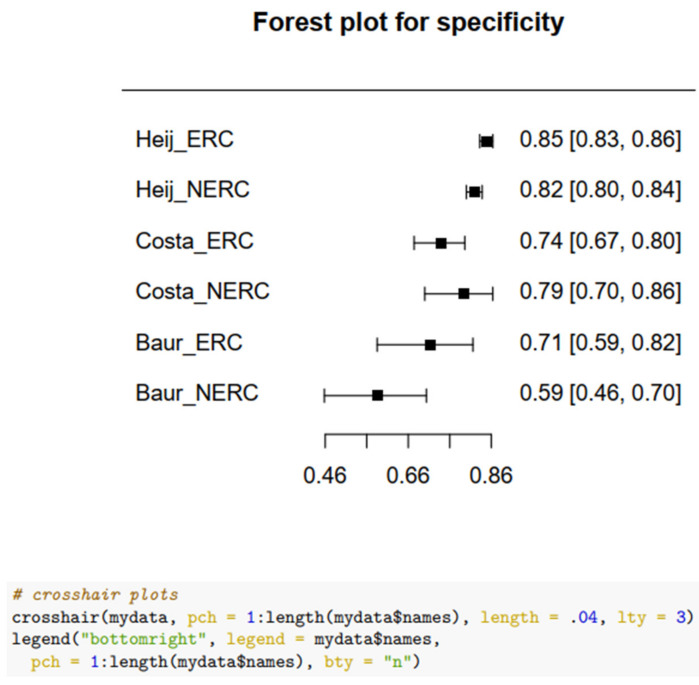
Forest Plot for Specificity.

For more information, see Figure 5: Forest Plot for Sensitivity; Figure 6: Forest Plot for Specificity.

**Figure 7:**
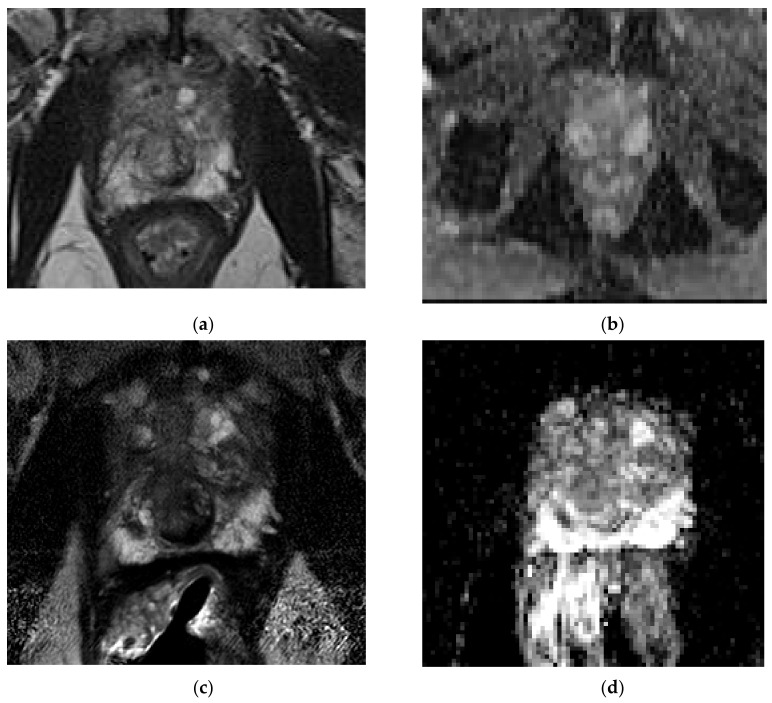
Small cancer not detected on NERC: a 71-year-old man, PSA = 8.7 ng/mL, Gleason 3 + 4 right posterior, lateral mid-gland peripheral zone cancer (arrow) not detected on NERC T2WI (**a**) and NERC ADC (**b**) exam, detected three months later as T2 hypo-intensity on Philips Achieva 3T ERC T2WI (**c**) and ERC ADC (**d**). Images courtesy of University of Chicago.

**Figure 8:**
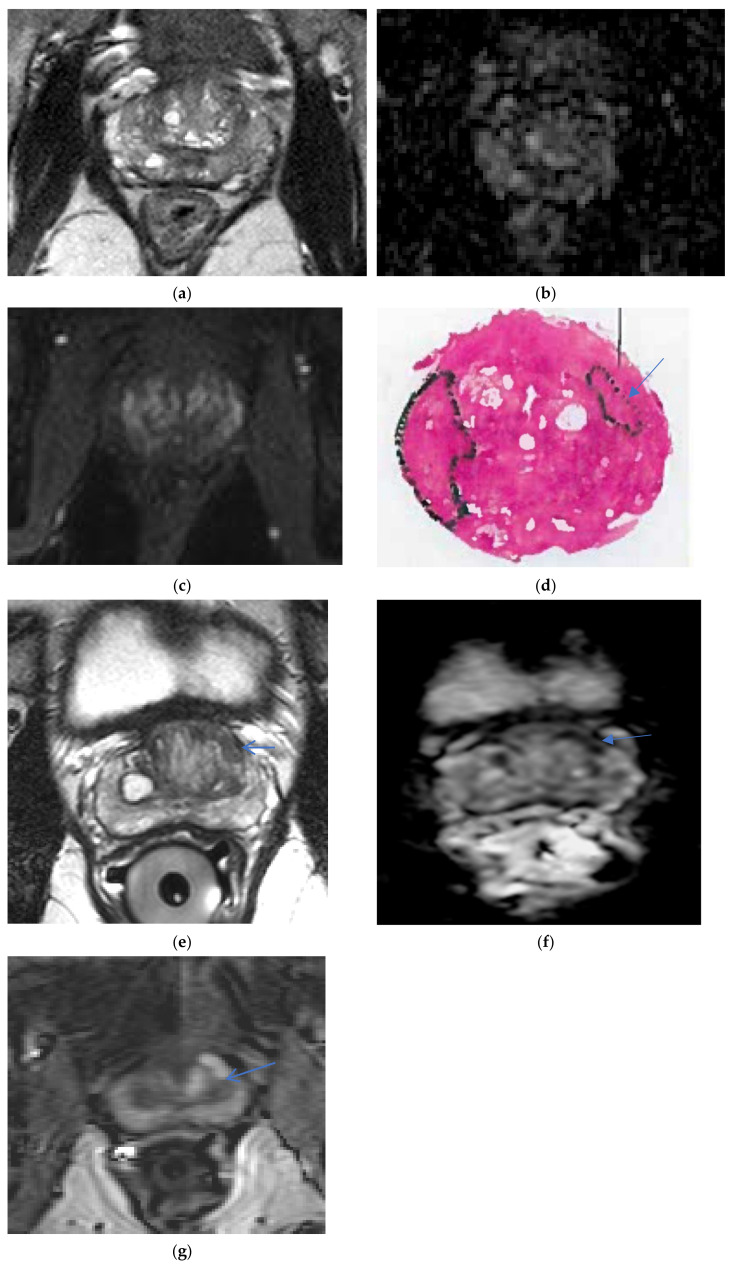
Small cancer not detected on NERC: a 64-year-old man, PSA = 4.2 ng/mL, Gleason 3 + 4 with extra-prostatic extension left mid-gland anterior peripheral zone missed on NERC T2WI (**a**), NERC ADC (**b**), and NERC DCE (**c**); detected (arrow) as T2 hypo-intensity on Philips Achieva 3T ERC T2WI (**e**), ERC ADC (**f**), and early enhancement on ERC DCE (**g**), with corresponding pathology (**d**). Images courtesy of University of Chicago.

The ERC-detected size of cancers was smaller compared to NERC, as concluded by Turkbey et al., Baur et al., Dhatt et al., and Gawlitza et al., Turkbey et al., found that the ERC mean lesion maximum diameter detection = 17.4 mm versus NERC mean lesion maximum size detection = 22 mm [15]. Baur et al. specifically found ERC had a smaller mean cancer maximum diameter detection on T2WI and DWI: for ERC T2WI, the mean maximum diameter detected lesion = 14.4 mm, 13.6 mm for two readers; for NERC T2WI, the mean maximum diameter detected lesion = 16.3 mm, 13.9 mm. For ERC DWI, the detected cancer maximum diameter = 15.1 mm, 14.3 mm. For NERC DWI, the detected cancer maximum diameter = 16.3 mm, 14.1 mm [12]. Gawlitza et al. [14] also found ERC lesion detection size to be smaller: average maximum diameter of detected lesion for ERC = 12.5 mm versus NERC = 13.5 mm [14]. ERC detected smaller lesions for the Gawlitza’s et al. less experienced reader, with the mean size of the ERC detected cancer diameter = 9.9 mm versus NERC detected cancer diameter = 11.9 mm [14]. Moreover, Turkbey et al. found the maximum diameter of missed lesions on ERC = 7.2 mm versus NERC = 9.2 mm [15] was smaller with ERC, also confirmed by Dhatt et al. [17], which concluded that NERC missed lesions equal to or larger than 10 mm, which was significant for active surveillance patients, but it was hoped that the misses would be detected by PSA monitoring [17]. Although Dhatt et al. [17] demonstrated equivalent image quality and diagnostic performance of both methods, NERC missed four cancers detected by ERC, with maximum diameters of 9, 10, 10, and 16 mm, attributing the false negatives to lower SNR of the NERC exam. ERC was better for detection of smaller cancer lesions, see Figure 7 and Figure 8.

**Figure 9:**
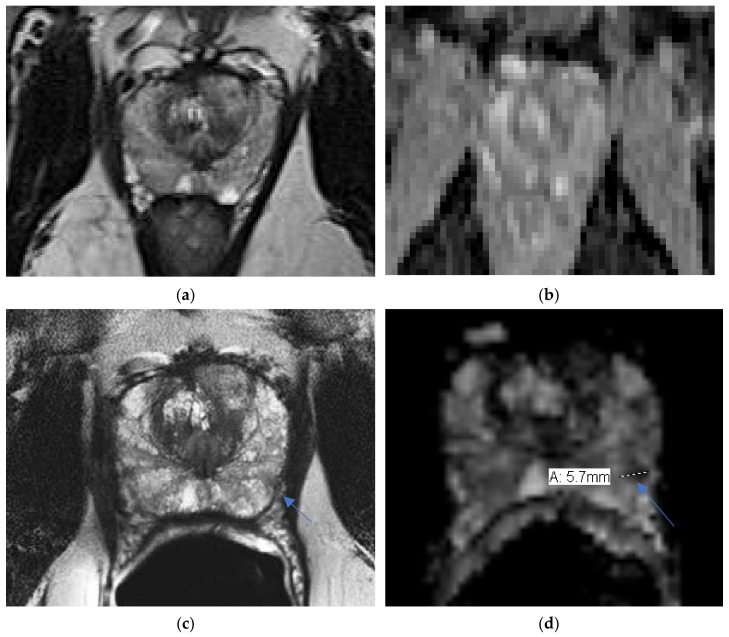
Small cancer accurately localized in the peripheral zone with ERC: a 47-year-old man, PSA = 7 ng/mL, with small left mid-gland posterior lateral peripheral zone Gleason 3 + 4 cancer (arrow) undetected on NERC T2WI (**a**) and NERC ADC (**b**) in a background of prostatitis, detected four months later with hypo-intense signal on Philips Achieva 3T ERC T2WI (**c**) and ERC ADC (**d**). Images courtesy of University of Chicago.

Early studies concluded higher ERC accuracy in localization of lesions with ERC AUC = 0.68 versus NERC AUC = 0.62, *p* < 0.001 [11]; ERC had higher detection accuracy of localization of peripheral zone lesions ERC AUC = 0.68 versus NERC AUC = 0.58, *p* < 0.001; and ERC had higher detection accuracy of localization of central gland lesions ERC AUC = 0.66 versus NERC AUC = 0.60, *p* < 0.001 [11]. Another study found that peripheral zone lesion detection was higher with ERC: ERC = 87% versus NERC = 76.5% [14]; there was no significant difference in transition zone lesion detection accuracy: ERC = 63% versus NERC = 61.5% [14]. More recent studies [18] in 2019, comparing 871 3T ERC versus 3T NERC exams with standard of reference of whole mount pathologic specimens from 2009–2016, demonstrated a higher detection rate with ERC. There was a higher ERC posterior gland detection rate, where cancers are more prevalent; ERC detection of posterior lesions = 58% versus NERC detection of posterior lesions = 48.1%, *p* = 0.025. There was also a higher ERC detection rate of peripheral zone cancers, with an ERC detection rate of peripheral zone cancer = 53.75% versus NERC detection rate of peripheral zone cancers = 45.2%, *p* = 0.033, where 70–80% of cancers are located. ERC can localize cancer lesions in the peripheral zone, see Figure 9. In one study, NERC missed four tumors, one of which was located in the TZ, a location which is difficult to detect [17].

ERC had a high sensitivity of detecting significant versus non-significant cancers, ERC detection of high-grade lesions = 84% versus NERC detection of high grade lesions = 76.5%, *p* = 0.106 [14]. When comparing ERC with NERC in the detection of high- and low-grade cancers, both methods were equivalent in detecting high grade lesions, but ERC had a higher detection rate for low grade lesions, especially for the more experienced reader [14]. ERC AUC of Gleason >/= 7 was 0.96 versus NERC AUC = 0.90. ERC detected 13/13 of Gleason 3 + 4 lesions, while NERC only detected 9/13 Gleason 3 + 4 lesions [17]. The ERC sensitivity of detection of Grade Group 2 (Gleason 3 + 4 = 7) = 93.3% and 86.7% (for two readers) versus NERC sensitivity of detection of Grade Group 2 = 76.7% and 83.3%, and this may carry some clinical significance of missing low-grade cancers. Specificity for ERC = 98.3 and 98.7% versus NERC = 98.7 and 98.7% was similar for Gleason Grades 3 and 4 detection for both readers [17]. Although Dhatt et al. [17] demonstrated equivalent image quality and diagnostic performance of both methods in detecting lesions of Gleason greater or equal to 4 + 3 (grade group 3 and higher), NERC missed four Gleason 3 + 4 cancers detected by ERC, with maximum diameters of 9, 10, 10, and 16 mm, with the false negatives attributed to lower SNR of the NERC exam.

**Figure 10:**
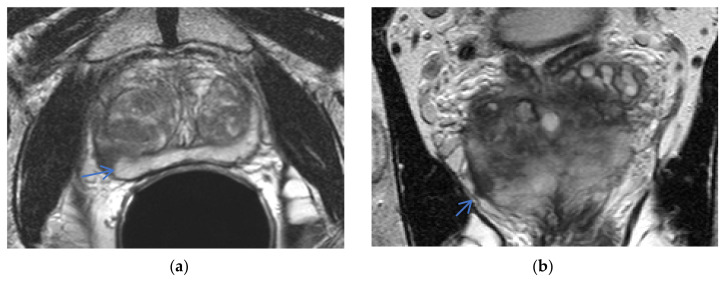

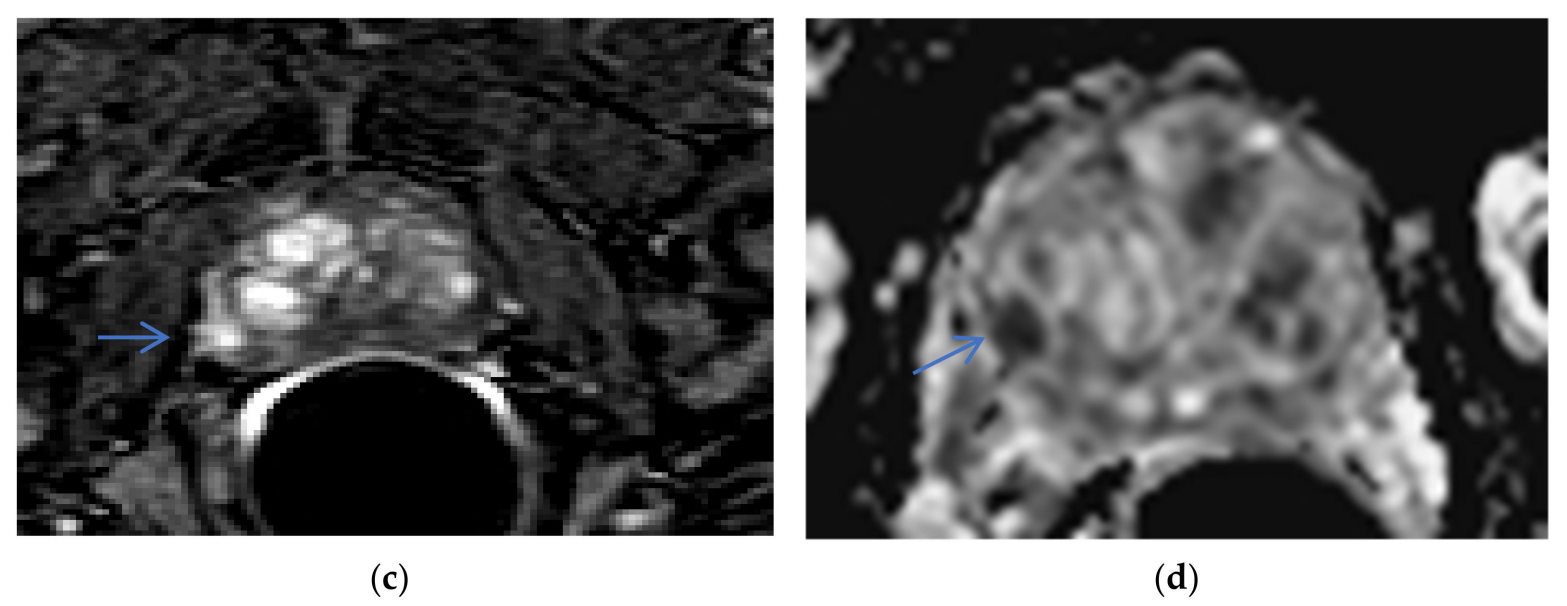
ERC demonstrates extra-prostatic extensions as little as 1.0 mm: a 56-year-old man with PSA = 6.7 ng/mL, right mid-gland peripheral zone Gleason 3 + 4 cancer (arrow) with extra-prostatic extension beyond the T2 hypo-intense line on axial ERC T2WI (**a**) and coronal ERC T2WI (**b**), ERC DCE (**c**) and ERC ADC (**d**) on Philips Achieva 3T. Images courtesy of University of Chicago.

**Figure 11:**
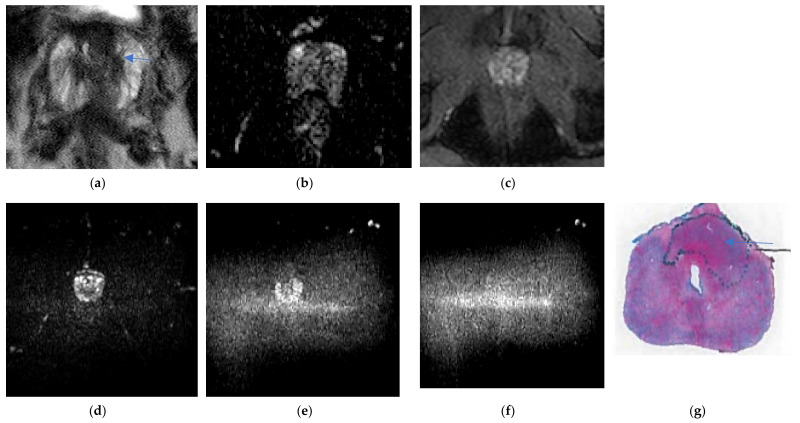
NERC unable to clearly identify extra-prostatic extension: a 64-year-old man, PSA = 7.2 ng/mL, Gleason 4 + 3 anterior mid-gland transition zone with extra-prostatic extension demonstrated as T2 hypo-intensity on Philips Achieva 3 T NERC T2WI (**a**), and indicated on whole mount pathology (**g**), undetected on NERC ADC (**b**), NERC DCE (**c**), NERC DWI b = 50 s/mm^2^ (**d**), NERC DWI b = 900 s/mm^2^ (**e**), NERC DWI b = 1500 s/mm^2^ (**f**). Images courtesy of University of Chicago.

ERC had a higher accuracy in staging cancer, with a higher detection of extra-prostatic extension and seminal vesical invasion; the sensitivity, specificity, and accuracy of detecting T3a, T3b, and T3 a and b cancers have been evaluated [4]. The ERC staging accuracy, sensitivity, specificity for seminal vesicle invasion = 83%, 46%, 92% versus NERC staging accuracy, sensitivity, specificity = 81%, 43%, 93%. For extra-prostatic extension, the staging accuracy, sensitivity, and specificity of ERC was 64%, 33%, 96%, versus NERC = 61%, 31%, 98%, in Kim et al. retrospective study [19]; although the statistics were equivalent in the Kim et al., study [19], the exams were not performed in a single sitting, and the methods are not directly comparable. Heijmink [11] in 2007 demonstrated improved accuracy in staging for three readers, ERC AUC = 0.91 versus NERC AUC = 0.63 [11]; ERC sensitivity for localized staging, extra-prostatic extension, and seminal vesical invasion ERC sensitivity = 73–80% versus NERC sensitivity = 7–13%; and ERC and NERC = 97–100% specificity were similar [11]. ERC can detect small areas of extra-prostatic extensions of cancer (see Figure 10), whereas NERC can miss areas of extra-prostatic extensions of cancer (see Figure 11).

## 5. Conclusions

In summary, controversial use of ERC at 3T is crucial for improving objective quality by providing increased signal to noise to the posterior and peripheral zone, where most cancers are located, and to the DWI and high b-value ADC sequences. ERC also increased contrast to noise. ERC subjectively improved quality by enhancing anatomic detail on T2WI, and somewhat enhanced anatomic detail on DWI, despite causing greater occurrences other artifacts on DWI. ERC caused less motion artifact on DWI than NERC DWI due to the stabilizing force of the coil, with other artifacts more prevalent on ERC DWI. ERC improved diagnostic performance with higher sensitivity and specificity with our pooled ERC sensitivity = 0.75 versus pooled NERC sensitivity = 0.65, pooled ERC specificity = 0.77 versus pooled NERC specificity = 0.74. The ERC PPV in the detection of overall and index lesions was higher than NERC PPV. ERC had a higher detection of smaller and less aggressive lesions, and had a smaller number of missed lesions. ERC had a higher accuracy than NERC in localizing and staging lesions.

**Figure 12:**
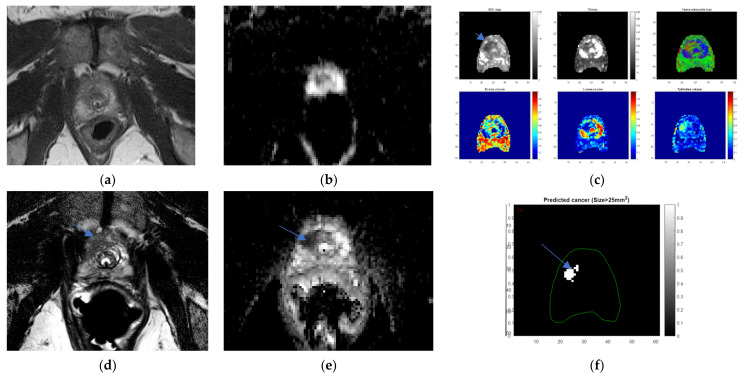
Hybrid color and risk maps (**c**,**f**) highlight cancer (arrow) not detected on NERC: a 58-year-old man, PSA = 3.2 ng/mL with right apex anterior transition zone Gleason 3 + 4 cancer undetected on Philips Achieva 3T NERC T2WI (**a**) and NERC ADC (**b**), detected six months later on Philips Achieva 3 T ERC T2WI (**d**) and ERC ADC (**e**), aided by hybrid risk map (**f**). Images courtesy of University of Chicago.

Future efforts to improve performance with enhanced signal to noise with better coils, such as the WPC, whose diagnostic performance has not yet been studied, are in development. Furthermore, it might be that different vendors will have different hardware or software performances, therefore not all 3T MRI machines will be alike, thereby influencing overall image quality and diagnostic performance. However, studies specifically comparing vendors are lacking, and therefore no conclusions can be drawn on this point. Performance can also be improved with quality monitoring algorithms. Diagnosis will also become more directed towards the stratification of lesions aided by artificial intelligence and deep learning techniques that automatically classify lesion aggressiveness by utilizing ADC and T2WI texture traits [20] or kurtosis models [21,22]. Additionally, microstructural quantitative imaging methods, such as luminal water imaging [23], HYBRID [24] (see Figure 12), and VERDICT [25], are evolving to identify foci of malignant tissue composition to achieve greater specificity and conspicuity of malignant lesions. These multifaceted efforts may obviate the need for the endorectal coil.

Prostate mpMRI continues to strive to improve the accuracy of detection, staging, and stratification of prostate cancer in a noninvasive, cost-effective manner. Wide availability, high quality exams, and cost containment tools are critical for providing a standardized system for both high quality image production and interpretation. Controversial utilization of the endorectal coil has long been debated in the necessity of providing accurate detection and staging of disease, encountering obstacles of patient dissatisfaction, time, cost, and coil artifacts for the most crucially diagnostic DWI sequences. Various strategies are underway to circumvent the need for the endorectal coil: mechanisms to enhance signal, resolution, conspicuity and characterization of high-grade prostate lesions with higher channel coils, malignant tissue modeling for deep learning, and microstructural quantitative imaging are being refined to produce efficient, quantitative imaging for the diagnosis, staging, and stratification of prostate cancer.

## Figures and Tables

**Table 1:** ^1^Study Characteristics.

Author	Year	Duration	Design-Prospective/ Retrospective	#Institutions	Consecutive Enrollment	Reference Standard
Heijmink [11]	2007	6/04–1/06	prospective	1	yes	prostatectomy
Kim [19]	2012	1/05–5/10	retrospective	1	yes	prostatectomy
Mazaheri [10]	2013	1/12–2/12	not recorded	1	not recorded	prostatectomy
Turkbey [15]	2014	3/10–9/10	prospective	1	yes	prostatectomy
Ha [8]	2014	2014	not recorded	not recorded	not recorded	not applicable
Costa [6]	2016	12/14–3/15	prospective	1	yes	prostatectomy or transrectal ultrasound biopsy
Baur [12]	2016	3/12–4/14	prospective	1	yes	transrectal ultrasound biopsy
Barth [13]	2016	11/14–3/15	prospective	2	not recorded	prostatectomy
Gawlitza [14]	2017	10/10–9/12	not recorded	1	not recorded	prostatectomy
Barth [16]	2019	12/14–8/15	retrospective	2	yes	prostatectomy
Mirak [18]	2019	7/09–12/16	prospective then retrospective	1	yes	prostatectomy
Dhatt [17]	2020	9/09–10/11	prospective	not recorded	not recorded	transrectal ultrasound biopsy then prostatectomy
Ullrich [9]	2020	6/17–4/19	retrospective	1	yes	transrectal ultrasound biopsy
O’Donohue [7]	2020	not recorded	prospective	1	yes	obturator internus muscle region of interest

^1^ Table 1: Study characteristics—the first author; year study was performed; length of the study; whether the study was prospective or retrospective; the number of institutions participating in the study; whether or not patients were consecutively enrolled in the study; and the reference standard used for comparison in the study are listed.

**Table 2:** ^1^ MRI Parameters.

Author	Slice Thickness DWI ERC	Slice Thickness DWI NERC	Slice Thickness DWI WPC	Slice Thickness T2 ERC	Slice Thickness T2 NERC	Slice Thickness T2 WPC	Matrix T2 ERC	Matrix DWI ERC	Matrix T2 NERC	Matrix DWI NERC	Matrix T2 WPC	NEX T2 ERC	NEX T2 NERC	NEX T2 NERC AUG	NEX T2 WPC	NEX DWI ERC	NEX DWI NERC	NEX DWI NERC AUG	NEX DWI WPC
Heijmink				2.5 mm	4.0 mm		76 × 384		512 × 512			1	2						
Kim				4.0/0.4 mm skip	4.0/0.4 mm skip		320 × 224		320 × 224			3	3						
Turkbey	3.0 mm	3.0 mm		3.0 mm	3.0 mm		304 × 242	not recorded	320 × 216	not recorded		not recorded	not recorded			not recorded	not recorded		
Ha				3.0 mm	3.0 mm	3.0 mm	528 × 528		528 × 528		528 × 528	2	2		2				
Mazaheri				3.0 mm	3.0 mm		128 × 128	not recorded	128 × 128	not recorded		2	2			not recorded	not recorded		
Costa ^2^																			
	Ingenia Scanner 2.5 mm	2.5 mm		3.0/0.3 mm skip	3.0/0.3 mm skip		400 × 289	128 × 121	400 × 289	128 × 121		1	1	2		2	2	4	
	Achieva Scanner 2.5 mm	2.5 mm		3.0/0.3 mm skip	3.0/0.3 mm skip		392 × 311	128 × 138	392 × 311	128 × 138		2	2	4		1	1	2	
Baur	3.0 mm	3.0 mm		3.0 mm	3.0 mm		0.6 × 0.6 mm resolution	1.6 × 1.6 mm resolution	0.6 × 0.6 mm resolution	1.6 × 1.6 mm resolution		not recorded	not recorded			not recorded	not recorded		
Barth	not recorded	not recorded		3.0 mm	3.0 mm		320 × 320	112 × 58	256 × 256	100 × 52		3	3			2, 4, 8	2, 6, 12		
Gawlitza	3.0 mm/ 0.6 mm skip	3.0 mm/ 0.6 mm skip		3.0 mm/ 0.6 mm skip	3.0 mm/ 0.6 mm skip		0.57 × 0.57 mm^2^	1.0 × 1.0 mm^2^	0.57 × 0.57 mm^2^	1.0 × 1.0 mm^2^		not recorded	not recorded			not recorded	not recorded		
Barth				3.0 mm	3.0 mm		320 × 320	112 × 58	256 × 256	100 × 52		3	4			2, 4, 8	2, 6, 12		
Mirak	3.6 mm	3.6 mm		3.0 mm	3.0 mm		320 × 310	160 × 94	320 × 310	160 × 94		2	2			8	8		
Dhatt				4.0 mm	4.0 mm		284 × 285	128 × 115	284 × 285	128 × 115		3	3			18	18		
Ullrich	not recorded	not recorded		3.0 mm		3.0 mm	384 × 384	128 × 64			320 × 300	1			1	6			6
O’Donohue	4.0 mm	4.0 mm	4.0 mm	3.0 mm	3.0 mm	3.0 mm	288 × 192	96 × 96	288 × 192	96 × 96	256 × 256	4	4		4	12	16		16

^1^Table 2: The first author; the MRI parameters of slice thickness of DWI for ERC, NERC, and WPC utilized; the slice thickness of T2WI for ERC, NERC, and WPC utilized; the matrix for DWI and T2WI for ERC; T2WI for NERC and T2WI WPC utilized; the NEX for T2 and DWI for ERC; NERC without and with augmentation; and WPC utilized. ^2^ MRI imaging parameters for two different Philips scanners, patients randomly assigned.

**Table 3:** ^1^ Patient Characteristics.

Author	Patients	Mean Age Years (Range)	Inclusion Criteria	Exclusion Criteria	Interval-Days (Range) Biopsy to MRI	Interval-Days (Range) MRI to Surgery	Mean PSA ng/mL (Range)
Heijmink	46	61 (51–70)	biopsy-proven clinically significant prostate cancer	contraindication to MRI, rectal coil, claustrophobia	112 (21–226)	14 (1–89)	7.8 (3.5–24.6)
Kim	151	64.8 (47–76) ERC	biopsy-proven prostate cancer, prostatectomy	contraindication to MRI, rectal coil, claustophobia, prior treatment, biopsy within 3 weeks of MRI	22.18 (22–45)	not recorded	11.69 (3–37)
		66.8 (55–76) NERC			27.65 (24–45)	not recorded	12.36 (4–38)
Ha	1 PHANTOM						
Mazaheri	25	49–76	transrectal ultrasound-guided biopsy proven prostate cancer	not recorded	not recorded	not recorded	not recorded
Turkbey	20	60.6 (50–74)	prostatectomy	no prostatectomy	not recorded	not recorded	6.8 (3.8–48.9
Costa	49	63 (49–79) ERC	clinical indication	no pathology, incomplete images,	37 (1–133)	same (and/or)	11.2 (2.5–48.5)
	23	63 (52–73) NERC-standard		claustrophobic, allergy to contrast	44 (5–118)	same (and/or)	7.9 (2.5–35.6)
	26	63 (49–79) NERC-augmented		contraindication to MRI or rectal coil	32 (1–133)	same (and/or)	14.1 (2.5–48.5)
Baur	45	66 (46–81)	elevated PSA,	claustrophobic, allergy	17 (1–107)	not recorded	12.3 (5.2–70)
			abnormal digital rectal exam,negative TRUS-biopsy	contraindication to MRI			
				contraindication to rectal			
				coil			
Gawlitza		64 (48–74)	biopsy-proven prostate cancer prior to prostatectomy	allergy to contrast	55 (11–119)	1.1 (1–3)	11.5 (0.6–56)
				contraindication to MRI or rectal coil			
Barth 2016	98	65.7 (42.21–78.1)	elevated PSA	prior surgery, radiation, incomplete exam, refused consent	not recorded	not recorded	5.7 (0.3–46)
			abnormal digital rectal exam				
Barth 2019	33	67.8 (51.3–77.3)	elevated PSA	no pathology	not recorded	85.5 (8–175)	5.8 (0.3–46)
			abnormal digital rectal exam				
Mirak	429	not recorded	within 6 months of prostatectomy, elevated PSA	prostate resection, radiation, metal hardware	not recorded	not recorded	7.9+/−9.0 (0.6–139)
Dhatt	22	64 (46–71)	TRUS-biopsy proven prostate cancer	contraindication to MRI, rectal coil	111.5 (64–151)	10 (4–18)	7 (6–8)
Ullrich							
	ERC = 150	67 (63–72)	biopsy-proven prostate cancer, or		not recorded	not recorded	5.9 (4.5–8.48)
	WPC = 66	66 (63–70)	elevated PSA		not recorded	not recorded	6.4 (4.4–8.4)
O’Donohue	18	63 (49–72)	biopsy-proven prostate cancer	no ERC, prostatectomy	not recorded	not recorded	10 (2–87)
			elevated PSA				

^1^ Table 3: Patient characteristics of number of patients; mean age years (range); inclusion criteria; exclusion criteria; interval days (range) biopsy to MRI; interval days (range) MRI to surgery; mean PSA (range) ng/mL.

**Table 4:** ^1^ Prostate Cancer Gleason Score, Location, and Presence/Absence of Extra-prostatic Extension.

Author	#Patients Gleason Score at Biopsy (Mean)							#Patients (Mean) Gleason Score at Surgery					#Patients Extraprostatic Extension/Total (%)	#Patients Seminal Vesical Invasion/Total (%)	#Lesions Anterior Prostate/Total (%)	#Lesions Posterior Prostate/Total (%)	#Lesions Transition Zone/Total (%)	#Lesions Peripheral Zone/Total (%)
	3 + 2	6	7 (3 + 4)	7 (4 + 3)	8 (4 + 4)	8 (5 + 3)	9	6	7(3 + 4)	7(4 + 3)	8	9						
Heijmink	Median = 6 (5–9)							Median =7 (5–9)										
Kim-ERC		35 (55.1)	18 (28.6)		9 (14.3)		1 (1.6)	19 (30.2)	28 (44.4)		13 (20.6)	3 (4.8)						
NERC		50 (56.8)	22 (25)		12 (13.6)		4 (4.5)	23 (26.1)	42 (47.7)		14 (15.9)	9 (10.2)						
Ha			Not applicable															
Mazaheri		not specified																
Turkbey								13	34		2	1	ERC4/5(80)				16/51(31)	35/51(69)
													NERC1/5(20)					
Costa		not specified											6/25(14)	4/25(16)		not specified		
Baur		3	3	2	5		1											
Gawlitza	1	14	17	5	4													
Barth-2016		not recorded																
Barth		1	14	11	3	1	1						11	7				
Mirak		399	317	103	29		24					ER	91/142(64.1)		66/184 (35.9)	200/345 (58)	41/110 (37.3)	225/419 (53.7)
												NERC	59/102(57.8)		76/157 (48.4)	89/185 (48.1)	62/114 (54.4)	103/228 (45.2)
Dhatt		5	14	2	1			5	12	4		1						
Ullrich																		
ERC		51	22	6	1		3											
WPC		17	11	5	2		1											
O’Donohue		not recorded																

^1^Table 4: Patient characteristics of number of patients with Gleason score at biopsy and at surgery; number of patients with extra-prostatic extension and seminal vesicle invasion; number of cancer lesions in the anterior or posterior prostate; number of cancer lesions in the transition zone or peripheral zone.

**Table 5:** ^1^ Index Test Characteristics.

Author	Same Sitting	#Patients ERC	#Patients NERC	#Patients WPC	Vendor ERC	Vendor NERC	WPC	Glucagon?	MRI Sequences	b-Value(s/mm^2^)	Planes	#Readers	Experience	Blinded	Independent or Consensus
Heijmink	yes	46	46		Siemens Trio	Siemens Trio		yes	T2		3	4	4 year, 2 year, 3 mo, 3 mo	yes	independent
Kim	no	88	63		GE Excite	GE Excite		yes	T2		3	2	10 year, 2 year	not recorded	consensus
Turkbey	yes	20	20		Philips Achieva	Philips Achieva 6 channel		not recorded	T2,DWI	not recorded	3	2	not recorded	yes	independent then consensus
Ha		1 phantom			Philips Achieva	Philips Achieva 6 channel	5 channel		T1		axial				
Mazaheri	yes	25	25		GE Discovery 8 channel	GE Discovery 8 channel		not recorded	DWI	0, 600, 1000, 1200, 1500	3	1	not recorded	not recorded	not recorded
Costa	yes	49	49		Philips Ingenia 6 channel	Philips Ingenia 6 channel		not recorded	T2, DWI	0–2000 ERC	axial	6		yes	independent
										0–1000 NERC	axial				
					Philips Achieva 6 channel	Philips Achieva 6 channel		not recorded		0–2000	axial				
Baur	yes	45	45		Siemens Skyra			no	T2, DWI, DCE	0, 100, 500, 1000	3	2	5 year, 9 year	yes	independent
									T1						
Barth	yes	98	98		Siemens Skyra 18, 32 channel	Siemens Skyra 18, 32 channel		not recorded	T2, DWI	100; 600; 1000	axial	2	4 year, 10 year	not recorded	independent
Gawlitza	+/−yes	41	41		Siemens Skyra 6 channel	Siemens Skyra 6 channel		yes	T2, DWI	50, 100, 800, 1500	axial, coronal	2	6 year, 2 year	no	independent
Barth	yes	33	33		Siemens Skyra 18 channel	Siemens Skyra 18 channel		not recorded	T2, DWI	0, 50, 1000, 1400	3	2	4 year, 10 year	yes	independent
Mirak	no	260	169		Siemens Skyra, Trio, Verio	Siemens Skyra, Trio, Verio		yes	T2, DWI	0, 100, 400, 800, calculated 1400	3D, axial, coronal	2//1	mos, 10–18 year//6 year	yes	independent
														yes	
Dhatt	yes	22	22		Philips Achieva	Philips Achieva		not recorded	T2, DWI	0, 600, 1800, extrapolated high b-value	axial, coronal	2	3 year, 13 year	yes	independent
Ullrich	no	100		50	GE Premier		Air	not recorded	T2, DWI	0, 600	3				
O’Donohue	yes	18		18	GE Discovery 32 channel		Procure	yes	T2, DWI	500, 1400	axial	2	4 year, 9 year	yes	not recorded

^1^Table 5: Index test characteristics: same sitting NERC and ERC; number of patients; vendor of MRI unit; use of glucagon; sequences evaluated; b-values used; number of planes imaged; number of readers; readers’ length of experience; consecutive enrollment; independent or consensus reading.

**Table 6:** ^1^ Outcomes Measured.

Author	Diagnostic Performance Outcome										Image Quality																	
	Extra-prostatic Extension, Seminal Vesicle Invasion	Lesion Detection	Sensitivity, Specificity, PPV, NPV, Accuracy					Diameter Lesion Detected	Diameter Lesion Missed	Accuracy of Location Lesion Detected	Imaging Traits										SNR				CNR	Integral Uniformity	Confidence	Discomfort
			Sensitivity	Specificity	PPV	NPV	Accuracy				Overall	Motion	Flare Artifact	Other Artifact	Anatomy	Architecture	Fat outline	Rectal Angle	Resolution	Lesion Clarity	SNR	ADC SNR	PZ SNR	TZ SNR				
Heijmink	x	x	x	x	x	x	x			x	x	x	x	x	x	x	x	x	x	x								
Kim			x																		x							
Mazaheri																						x	x	x				
Turkbey			x		x			x	x																			
Ha																					x					x		
Costa			x	x			x	x	x																		x	
Baur			x	x				x	x		x	x	x	x	x	x	x	x										x
Barth 2016											x	x		x	x				x	x	x							
Gawlitza	x	x	x	x				x	x	x		x		x	x	x	x	x		x							x	
Barth 2019		x	x		x																							
Mirak		x	x	x						x																		
Dhatt		x	x	x				x	x	x	x				x	x				x		x						
Ullrich											x	x			x						x				x			
O’Donohue											x	x	x	x	x		x	x			x							

^1^Table 6: Outcomes measured: diagnostic performance parameters of extra-prostatic extension; lesion detection; significant lesion detection; sensitivity, specificity, PPV, NPV, accuracy; diameter lesion detected; diameter lesion missed; accuracy of location lesion detected; quality parameters of image quality; SNR; CNR; integral uniformity; confidence level; discomfort.
